# Comparative analysis of routes of immunization of a live porcine reproductive and respiratory syndrome virus (PRRSV) vaccine in a heterologous virus challenge study

**DOI:** 10.1186/s13567-016-0331-3

**Published:** 2016-03-17

**Authors:** Kang Ouyang, Jagadish Hiremath, Basavaraj Binjawadagi, Duan-Liang Shyu, Santosh Dhakal, Jesus Arcos, Rose Schleappi, Lynette Holman, Michael Roof, Jordi B. Torrelles, Gourapura J. Renukaradhya

**Affiliations:** Food Animal Health Research Program (FAHRP), OARDC, Department of Veterinary Preventive Medicine, The Ohio State University, Wooster, OH 44691 USA; College of Animal Science and Technology, Guangxi University, Nanning, China; Department of Microbial Infection and Immunity, The Ohio State University, Columbus, OH USA; Kalmbach Swine Management, L.L.C., Upper Sandusky, OH 43351 USA; Boehringer Ingelheim Vetmedica, Inc., Ames, IA USA

## Abstract

Porcine reproductive and respiratory syndrome (PRRS) is caused by PRRS virus (PRRSV), which infects primarily the respiratory tract of pigs. Thus intranasal (IN) delivery of a potent vaccine-adjuvant formulation is promising. In this study, PRRS-MLV (VR2332) was coadministered ± an adjuvant *Mycobacterium vaccae* whole cell lysate or CpG ODN through intramuscular (IM) or IN route as a mist, and challenged with a heterologous PRRSV 1-4-4 IN at 42 days post-vaccination (dpv). At 14 and 26 dpv, vaccine viral RNA copies were one log greater in the plasma of PRRS-MLV IM compared to IN vaccinated pigs, and the infectious replicating vaccine virus was detected only in the IM group. In PRRS-MLV ± adjuvant IM vaccinated pigs, reduced viral RNA load and absence of the replicating challenged virus was observed at 7, 10 and 14 days post-challenge (dpc). At 14 dpc, in BAL fluid ≥5 log viral RNA copies were detected in all the pig groups, but the replicating challenged virus was undetectable only in IM groups. Immunologically, virus neutralizing antibody titers in the plasma of IM (but not IN) vaccine groups was ≥8 against the vaccine and challenged viruses. At 26 dpv, PRRS-MLV IM (without adjuvant) received pigs had significantly increased population of CD4 and CD8 T cells in PBMC. At 14 dpc, relatively increased population of IFN-γ^+^ total lymphocytes, NK, CD4, CD8 and γδ T cells were observed in the MLV-IM group. In conclusion, PRRS-MLV IM vaccination induced the virus specific T cell response in pigs, but still it is required to improve its cross-protective efficacy.

## Introduction

Porcine reproductive and respiratory syndrome (PRRS) is a respiratory disease of pigs of all ages and a reproductive disease of sows. PRRS is an endemic disease in the swine industry worldwide [1], and causes approximately $664 million losses annually in the USA [2]. PRRS virus (PRRSV) is the causative agent, isolated simultaneously in Europe and North America in the 1990s [3, 4]. PRRSV belongs to the *Arteriviridae* family [5] and displays a high genetic diversity [6]. To control PRRS, a modified live-attenuated PRRS vaccine (PRRS-MLV) has been widely used since the 1990s. PRRS-MLV protects pigs from clinical disease with reduced lung lesions [7] and viral shedding [8], and elicits a protective response against homologous virus. But apart from concerns about safety of PRRS-MLV in vaccinated pigs [9, 10], the breadth of cross-protection induced by MLV is highly questionable [11, 12].

Adjuvants are necessary to potentiate vaccine efficacy. Vaccine inoculated to a mucosal site along with a potent adjuvant upregulates the expression of costimulatory molecules on immune cells, which secrete chemokines and cytokines [13]. Mucosal vaccine coadministered through potent adjuvant/s induces superior cross-protective immunity by enhancing the array of antigen specific T and B cell responses; mediated by dramatic increase in spreading of antigenic epitopes and recognition of multiple conserved epitopes, which otherwise are not recognized [14–17]. Thus, the potency of adjuvant and delivery system determine the degree of cross-protection. PRRSV causes disease primarily in the respiratory tract and thus intranasal (IN) delivery of PRRS-MLV with a potent adjuvant is promising.

We have demonstrated that pigs vaccinated with PRRS-MLV intranasally with a potent adjuvant, *Mycobacterium tuberculosis* whole cell lysate (*M. tb* WCL), elicits better cross-protective immune response against heterologous challenge than without adjuvant [16]. But large-scale production of *M. tb* WCL involves risk, time and cost, as *M. tb* is a Biosafety Level (BSL)-3 agent. Alternatively, a non-pathogenic *Mycobacterium* (*M. vaccae*) adjuvant was shown to stimulate cytotoxic T-cell response [18] and provides protective response against tuberculosis [19]. Another vaccine adjuvant, CpG-oligodeoxynucleotide (CpG-ODN), activates the professional antigen-presenting cells [20] and potentiates the antibody and cross-reactive T cell response in mice [21, 22] and pigs [23, 24].

Therefore, in this study we evaluated cross-protective efficacy of PRRS-MLV coadministered with adjuvant *M. vaccae* WCL or CpG ODN through the IM or IN route. Our results suggest that those two adjuvants are not potent enough to augment the breadth of immunity against PRRS; rather PRRS-MLV delivered IM without any adjuvant is relatively better. Our results suggest that further studies are required to improve the cross-protective efficacy of PRRS-MLV using other highly potent adjuvants delivered IM and IN.

## Materials and methods

### Cells, PRRSV and adjuvant

MARC-145 cells were used for growing PRRSV [25] and in immunological assays [26]. Cells were maintained in high glucose DMEM (HyClone, MA, USA) supplemented with 0.1 mM HEPES (Fisher Scientific, NJ, USA), antibiotic/antimycotic solution (HyClone, UT, USA) and 10% FBS (Atlanta Biologicals, GA, USA) at 37 °C in a humidified atmosphere with 5% CO_2_. DMEM containing 2% FBS was used to grow virus in MARC-145 cells. PRRS-MLV was provided by Boehringer Ingelheim^®^. A field PRRSV strain 1-4-4 isolated from infected sows in Ohio was used to challenge pigs. PRRS-MLV parent strain VR2332 and a genetically variant Type 2 PRRSV strain MN184 [27] were used to analyze the virus specific neutralizing antibody (NA) titers in plasma and BAL fluid samples.  PRRSV ORF5 nucleotide sequence similarity between vaccine strain VR2332 and challenge stain 1-4-4 is 85.6%. All the PRRSV were propagated in MARC-145 cells and aliquots were stored at −80 °C until use.

*Mycobacterium vaccae* (ATCC#23027) was grown in endotoxin free 7H9 medium at 37 °C in accordance with ATCC instructions, and the whole cell lysate (WCL) was prepared as previously described [28]. CpG ODN 2007 (TCGTCGTTGTCGTTTTGTCGTT) in phosphorothioate backbone was custom prepared (Integrated DNA Technologies, IA, USA). Five conserved T cell peptides of PRRSV, nsp10 (aa 2578-2628) CPGKNSFLDEAAYCNHL and (aa 2554-2604) VRILAGGWCPGKNSFLD; Nsp9 (aa 442-492) VRGNPERVKGVLQNTRF [29]; GP5 (aa 445-489) KGRLYRWRSPVIIEK [30]; and N (aa 187-213) VRHHFTPSE [31] were custom synthesized (Thermofisher Scientific, NY, USA).

### Pigs and inoculation

**Figure 1:**
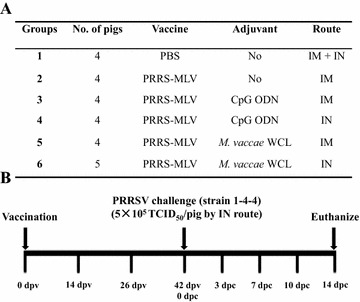
**PRRS-MLV vaccination and challenge study work plan.** **A** Conventional crossbred pigs were randomly divided into six Groups (4 or 5 per Group). PRRS-MLV was administered once without (saline) or with an adjuvant, *M. vaccae* WCL or CpG ODN, through intramuscular (IM) or intranasal (IN) route. **B** All the experimental pigs were challenged with a heterologous PRRSV strain 1-4-4 by IN route at 42 dpv. Blood samples were collected and rectal temperature and body weight were recorded at 0, 14, 26, 42 dpv and 3, 7, 10, 14 dpc and pigs were euthanized at 14 dpc.

A total of 25 conventional Large White-Duroc crossbred 4 week old pigs were procured from The Ohio State University swine herd, which were seronegative for PRRSV, porcine respiratory coronavirus, transmissible gastroenteritis virus and porcine circovirus 2. Pigs were allowed to acclimate for a week before initiation of the experiment, and randomly divided into six groups (*n* = 4 or 5 per group) (Figure 1A). Animals were maintained with food and water ad libitum in our large animal BSL2 facility under the supervision of a veterinarian. The adjuvant (*M. vaccae* WCL 5 mg/pig or CPG-ODN 250 μg/pig) and PRRS-MLV (the same vaccine dose as per the manufacturer’s recommendation was inoculated either by IM or IN route) were not mixed for inoculation in pigs; and they were inoculated separately on either side of the neck through IM or IN to both nostrils as mist. The pig group vaccinated intranasally with PRRS-MLV without adjuvant was not included in this study, but such a group was included in our previous study [16]. Each pig received 2 mL vaccine formulation once, with 1 mL of vaccine/adjuvant to each side of the neck or each nostril. Pigs were challenged using a heterologous PRRSV strain 1-4-4 (5 × 10^5^ TCID_50_ per pig) on 42 days post-vaccination (dpv) IN as mist and euthanized on 14 days post-challenge (dpc) (Figure 1B). Rectal temperature and body weight of pigs were recorded during the experiment on the days of blood collection. Collection of samples, maintenance and euthanization of pigs were performed as per the approved animal use and care committee protocol of The Ohio state University.

### Collection of blood samples and bronchoalveolar lavage fluid of pigs

For evaluation of viremia and PRRSV specific neutralizing antibody response, 5–7 mL of blood was collected at 0, 14, 26, 42 dpv and 3, 7, 10, 14 dpc. Plasma was separated using K2 EDTA plus blood collection tubes (BD vacutainer, NJ, USA) and preserved at −80 °C until use in the assays. At 26 dpv and 14 dpc, peripheral blood mononuclear cells (PBMC) were isolated from blood collected in EDTA by gradient centrifugation using lymphoprep solution in sepmate tubes (Stem Cell Technologies, Canada) [32]. PBMC were resuspended in enriched-RPMI (E-RPMI, RPMI containing 10 % FBS, 200 μm HEPES, 1 mM sodium pyruvate, 25 μm 2-ME, 1× Non-Essential Amino Acid, and 1× antibiotic and antifungal). Bronchoalveolar lavage (BAL) fluid was collected during necropsy as described previously [33] and aliquots were stored at −80 °C until use in the assays.

### Virus and virus neutralizing antibody (NA) titration

Analysis of PRRSV and NA titers in plasma and BAL fluids were performed by indirect immunofluorescence assay (IFA) [34, 35]. Briefly, for virus titration confluent monolayer of MARC-145 cells in 96-well microtiter plate was treated with 10-fold dilution of samples for 48 h; for VNT, samples were heat (56 °C for 30 min) and UV (254 nm for 45 min) inactivated before use in the assay. Twofold serially diluted samples were incubated with equal volume of PRRSV (strain 1-4-4 and VR2332, 100 TCID_50_ per well; strain MN184, 200 TCID_50_ per well) for 1 h at 37 °C, and the mixture was transferred into microtiter plates containing a confluent monolayer of MARC-145 cells and incubated for 48 h at 37 °C. Cell plates were fixed using acetone-Milli-Q water (8:2) mixture for 10 min at room temperature (~20 °C) and dried. Plates were treated with anti-PRRSV nucleocapsid protein specific monoclonal antibody (SDOW17) (Rural Technologies Inc., SD, USA) (1:10 000) for 2 h at 37 °C, followed by Alexa Fluor 488 conjugated goat anti-mouse IgG (H + L) (Invitrogen, CA, USA) secondary antibody (1:4000). The plates were examined under a fluorescent microscope after mounting with glycerol-phosphate-buffered saline (PBS) (6:4). The virus induced cytopathic effect was examined under a fluorescent microscope and the titer was calculated using the Reed and Muench method [34]. PRRSV titer was expressed as the 50% tissue culture infective dose (TCID_50_) per mL. NA titer was the reciprocal dilution of the plasma or BAL fluid which caused >90% reduction in virus-specific fluorescence foci compared to virus control well.

### Detection of PRRSV RNA load by qRT-PCR

Plasma and BAL fluid samples were used in preparation of RNA by the MagMAX™ 96 viral RNA isolation kit (Ambion/Applied Biosystems, CA, USA) [33]. RNA isolation was performed using 50 µL of sample using the MagMAX™ Express Magnetic Particle Processor (Applied Biosystems, NY, USA) according to the manufacturer’s instructions. RNA was reverse transcribed into complementary DNA (cDNA) using Quantitect reverse transcription kit (Qiagen, Germany). The cDNA was used in real-time PCR reaction with PerfeCta SYBR Green Fast Mix (Quanta Biosciences, MD, USA) using the PRRSV ORF6 gene derived forward primer (GATAACCACGCATTTGTCGTC) and reverse primer (TGCCGTTGTTATTTGGCATA). Standard curves were generated using serial dilution of known copies of the PCR product. PRRSV RNA copies in each mL of plasma and BAL fluid were determined.

### Flow cytometric study of immune cell population

Flow cytometry analysis was performed to determine the population of lymphocyte subsets from 50 000 acquired events of immunostained PBMC [26, 33]. Briefly, PBMC isolated at 26 dpv and 14 dpc were plated (5 × 10^6^ cells/well) in 48-well tissue culture plate in the presence of PRRSV strain 1-4-4 (5 × 10^4^ TCID_50_/well, 0.01 MOI) or pooled five PRRSV conserved peptides (1 μg/mL of each peptide) in 1 mL E-RPMI for 48 h at 37 °C with 5% CO_2_, and cells cultured with E-RPMI medium only were included as a control. GolgiStop protein transport inhibitor (BD Bicoscience, CA, USA) and Goldiplug Brefeldin A (Sigma-aldrich, MO, USA) were added at the last 6 h of incubation [36, 37]. Cells were first surface-labeled using pig specific mAb (CD3ε, CD4α, CD8α, δ chain) conjugated with different fluorochromes [32, 38]. Subsequently, cells were fixed, permeabilized and stained for intracellular IFN-γ using fluorochrome-conjugated mouse anti-pig IFN-γ mAb or its isotype control mAb (BD Biosciences, CA, USA). Immunostained cells were acquired using the BD Aria II flow cytometer and analyzed using the FlowJo software.

### Ethics statement

This study was carried out in strict accordance with the recommendations by Public Health Service Policy, United States Department of Agriculture Regulations, the National Research Council’s Guide for the Care and Use of Laboratory Animals, and the Federation of Animal Science Societies’ Guide for the Care and Use of Agricultural Animals in Agricultural Research and Teaching. All the relevant institutional, state and federal regulations and policies were followed regarding care and use of animals at the Ohio State.

### Statistical analysis

All data were expressed as the mean value of 4 or 5 pigs ± SEM. Statistical analyses were performed using the GraphPad Prism 6 software by applying one way ANOVA followed by the Tukey’s *t*-test. The statistical significance was considered at *P* < 0.05.

## Results

### Clinical outcome and PRRSV viral load in pigs

**Figure 2:**
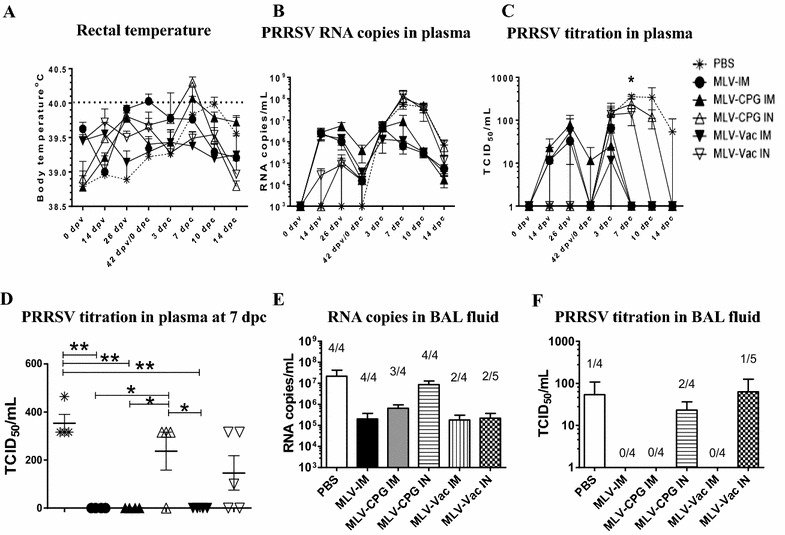
**Dynamics of body temperature and PRRSV load in the plasma and BAL fluid of pigs.** **(A)** Rectal temperature of pigs was recorded at the indicated dpv and dpc. PRRSV RNA copies and replicating infectious virus load in the plasma (**B**–**D**) and BAL fluid (**E**, **F**) were quantified by qRT-PCR and indirect immunofluorescence assay. The numbers above the bar in the graphs (**E**, **F**) represent the ratio of PRRSV positive pigs to the total in each group. Each data point or bar in the graph represents the average value from 4 or 5 pigs ± SEM. Asterisk marked in panel **C** indicates the virus titers in MLV-IM, MLV-CPG IM and MLV-IM Groups were significantly reduced compared to mock (PBS) challenged Group (*P* < 0.01) and MLV-CPG IN Group (*P* < 0.05) at 7 dpc. In panel **D** asterisks (*P < 0.05 and **P < 0.01) indicate statistical significant difference between the marked two pig Groups at 7 dpc.

Although we did not observe any respiratory distress in the experimental pigs, Group 3 (MLV-CPG IM), Group 4 (MLV-CPG IN) and Group 1 (PBS) pigs had moderate fever (>40 °C) at 7 and 10 dpc (Figure 2A). PRRSV titer (TCID_50_/mL) and RNA load (RNA copies/mL) in the plasma and BAL fluid were determined by qRT-PCR for PRRSV ORF6 and using MARC-145 cells for replicating infectious virus titers, respectively. In plasma, PRRSV RNA copies in Group 2 (MLV-IM), Group 3 (MLV-CPG IM) and Group 5 (MLV-Vac IM) pigs were 1-2 log higher compared to Group 4 (MLV-CPG IN) and Group 6 (MLV-Vac IN) at 14 and 26 dpv; and were 1-2 log lower at 7 and 10 dpc compared to Groups 4 and 6 (Figure 2B). In pre-challenged pigs, the replicating vaccine virus was detectable only in IM but not IN vaccinated groups (Figure 2C). At 7 dpc, detectable infectious virus in all the IM pig groups was absent and the data were statistically significant compared to pig Group 1 (PBS) (*P* < 0.01) and Group 4 (MLV-CPG IN) (*P* < 0.05) (Figure 2D). The replicating virus was undetectable in the plasma of Group 4 (MLV-CPG IN) at 14 dpc and at 10 and 14 dpc in Group 6 pigs (Figure 2C).

In the BAL fluid, ≥5 log viral RNA copies were detected in all the pig Groups; in particular, all four pigs of Group 1 (PBS) and Group 4 (MLV-CPG IN) had high levels of RNA copies (≥7 logs/mL) with 1-2 log greater than the other groups (Figure 2E). The replicating virus was absent in BAL fluid of all the IM pig groups, while one or two pigs in both the IN vaccinated groups still had infectious virus (Figure 2F).

### Virus neutralizing antibody level in plasma and BAL fluid

**Figure 3:**
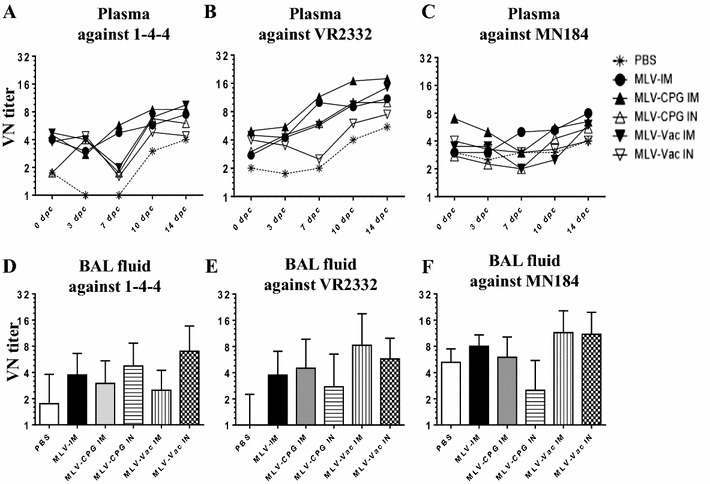
**PRRSV neutralizing antibody (NA) titers in the plasma and BAL fluid of pigs.** Viral NA titers in the plasma (**A**–**C**) and BAL fluid (**D**–**F**) against challenged PRRSV strain 1-4-4 (**A**, **D**), PRRS-MLV parent strain VR2332 (**B**, **E**) and a genetically variant Type 2 PRRSV strain MN184 (**C**, **F**) were determined by indirect immunofluorescence assay. The NA titer of a sample was expressed as the reciprocal of the highest dilution that inhibited >90% of the virus induced immunofluorescence activity. Each data point or bar in the graph represents the average NA titer from 4 or 5 pigs ± SEM.

PRRSV-specific NA titers in the plasma and BAL fluid against strains 1-4-4, VR2332 and MN184 were analyzed. In plasma, the NA titers against strains 1-4-4 and VR2332 were gradually increased in all the pig groups, and in IM vaccine groups the titers were two-fold higher than IN vaccine groups with mean titer ≥1:8 at 10 and 14 dpc (Figures 3A and B). In contrast, NA titer against strain MN184 remained <1:8 in all the tested pig groups (Figure 3C). In BAL fluid, the NA titers against the three tested viral strains were <1:8 in most of the pig groups, and in Groups 5 and 6 against MN184 strain the NA titers were >1:8 (Figures 3D–F).

### Phenotypic analysis of different T lymphocyte subpopulations in pigs

**Figure 4:**
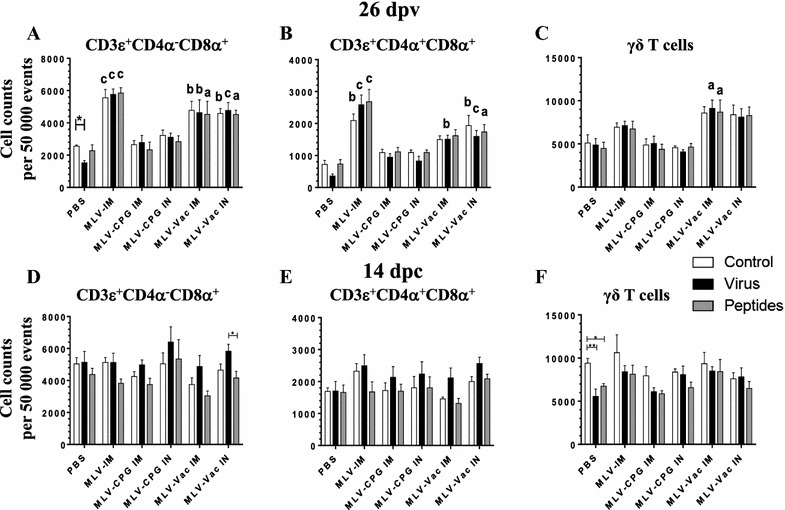
**Population of PRRSV specific lymphocyte subsets in the blood of pigs.** PBMC isolated from individual pigs were unstimulated (control) or stimulated with PRRSV (virus) or pooled five conserved peptides (peptides) and immunostained using indicated pig specific cell surface markers and analyzed by flow cytometry. Population of CD3ε^+^CD4α^−^CD8α^+^, Th/memory cells (CD3ε^+^CD4α^+^CD8α^+^) and γδ T cells in the PBMC collected at 26 dpv (**A**–**C**) and 14 dpc (**D**–**F**) are shown. Each bar represents the average cell count of 50 000 events from 4 or 5 pigs ± SEM. Lowercase alphabets and asterisks indicate a statistically significant difference (*a* or *P < 0.05, *b* or **P < 0.01 and *c* *P* < 0.001) between control (PBS) and vaccine received pig groups under the same ex vivo stimulation condition, or between different stimulation conditions within the same treatment group.

The population of CD3ε^+^CD4α^−^CD8α^+^ cells and T-helper/memory cells (CD3ε ^+^CD4α^+^CD8α^+^) in PBMC of Group 2 (MLV-IM), Group 5 (MLV-Vac IM) and Group 6 (MLV-Vac IN) pigs at 26 dpv was significantly increased compared to Group 1 (PBS) (Figures 4A and B). Only MLV-Vac IM pig group had increased counts of γδ T cells compared to PBS control (Figure 4C). The population of CD3ε^+^CD4α^−^CD8α^+^ cells was significantly increased in the MLV-Vac IN pig group restimulated with virus compared to peptide stimulation at 14 dpc (Figure 4D).

**Figure 5:**
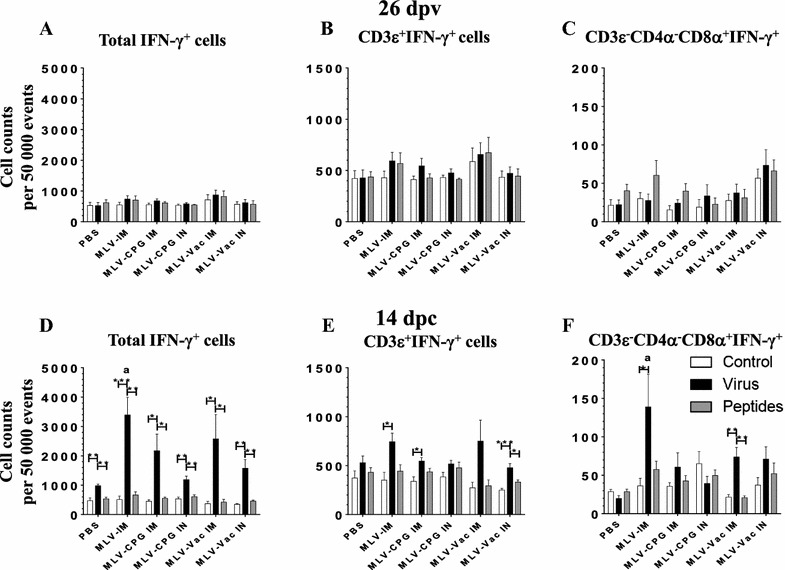
**Population of PRRSV specific IFN-γ** ^**+**^ **total lymphocyte response in the blood of pigs.** PBMC isolated from individual pigs at 26 dpv (**A**–**C**) and 14 dpc (**D**–**F**) were unstimulated (control) or stimulated with PRRSV strain 1-4-4 (virus) or pooled five conserved peptides (peptides). IFN-γ secreting cells in total CD3ε^+^ lymphocytes and NK cells (CD3ε^−^CD4α^−^CD8α^+^) are shown. Each bar represents the average cell counts of 50 000 events from 4 or 5 pigs ± SEM. Lowercase alphabet and asterisks indicate a statistically significant difference (*a* or *P < 0.05, **P < 0.01 and *** *P* < 0.001) between control (PBS) and vaccine received groups under the same ex vivo stimulation condition, or between different stimulation conditions within the same treatment group.

**Figure 6:**
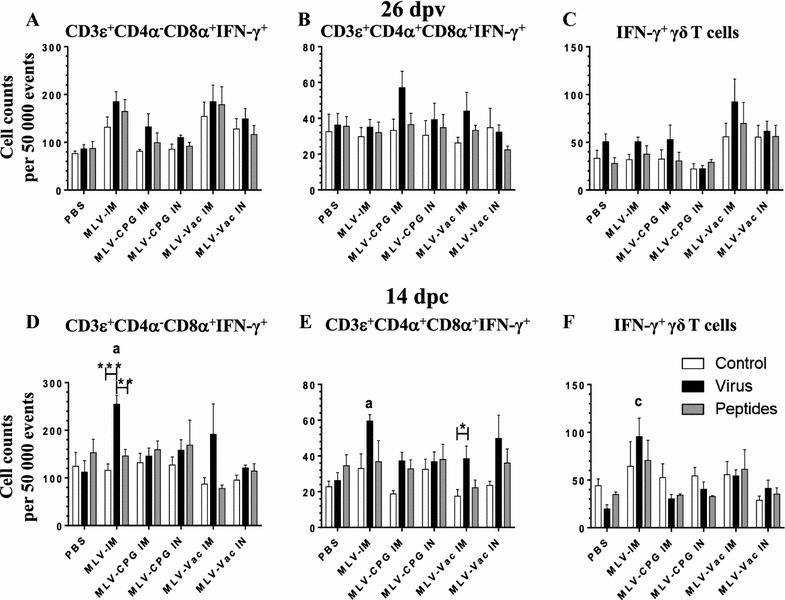
**PRRSV specific IFN-γ** ^**+**^ **T lymphocyte subsets in the blood of pigs.** PBMC isolated from individual pigs at 26 dpv (**A**–**C**) and 14 dpc (**D**–**F**) were unstimulated (control) or stimulated with PRRSV (virus) or pooled five conserved peptides (peptides). Cells were immunostained for T cell surface markers CD3ε, CD4α, CD8α, and δ chain (γδ T cells) and intracellular IFN-γ^+^ cells are shown. Each bar represents the average cell counts of 50 000 events from 4 or 5 pigs ± SEM. Lowercase alphabet and asterisks indicate a statistically significant difference (*a* or *P < 0.05, **P < 0.01 and *c* or ***P < 0.001) between control (PBS) and vaccine received groups under the same ex vivo stimulation condition, or between different stimulation conditions within the same treatment group.

At 26 dpv, none of the pig groups had increased activated (IFN-γ^+^) lymphocyte subsets (Figures 5A–C and 6A–C). But at 14 dpc, significantly increased population of total and CD3ε^+^ IFN-γ secreting cells and IFN-γ^+^ CD3ε^−^CD4α^−^CD8α^+^ (NK) cells were detected (Figures 5D–F). Also, IFN-γ positive CD3ε^+^CD4α^−^CD8α^+^ (cytotoxic T cell (CTL)/γδ T cell), CD3ε^+^CD4α^+^CD8α^+^ (T-helper/memory) and γδ T cells were observed in Group 2 (MLV-IM) pigs restimulated with the challenge virus ex vivo compared to control and other vaccine trial groups (Figures 6D–F).

## Discussion

The restriction fragment length polymorphism (RFLP) code for the Type 2 prototype PRRSV strain VR2332 derived PRRS-MLV (Boehringer Ingelheim^®^) was 2-5-2 [39], and it was passaged repeatedly in MARC-145 cells and became avirulent in pigs [32]. The RFLP code of the challenged field virus used in this study was 1-4-4, and it was widely prevalent in North America and has approximately 15% difference in amino acid sequence compared to VR2332 GP5 sequence [40]. Thus, we analyzed the efficacy of PRRS-MLV ± *M. vaccae* WCL or CPG ODN administered IM or IN against the genetically heterologous viral strain 1-4-4 in pigs.

We previously demonstrated that pigs immunized with PRRS-MLV with *M. tb* WCL IN elicit cross-protective response against a heterologous PRRSV strain MN184, which also has 15% nucleotide difference in GP5 sequence compared to VR2332 [16]. In this study, our results indicated that PRRS-MLV IM with and without adjuvant received pigs had 1-2 log reduced challenged viral RNA compared to IN route vaccinated animals. Further, in all the IM vaccinated pigs the replicating challenged virus was absent at 7, 10 and 14 dpc in plasma and also in BAL fluid at 14 dpc. Immunologically, these results were supported with NA titers of >1:8 against the strain 1-4-4 in IM vaccinated pigs. This data was consistent with an earlier study wherein PRRSV NA titer of 1:8 or higher protects piglets against development of viremia [41, 42]. In this study, IM vaccinated pigs with a single dose of PRRS-MLV had partial protection to a heterologous challenge virus. Similar degree of partial protection was observed in a heterologous challenge study using a highly pathogenic PRRSV strain JX143 derived PRRS-MLV vaccinated pigs [43].

The protective response against PRRSV in PRRS-MLV vaccinated pigs was found to be dependent more on the levels of cell-mediated than humoral immunity [44]. In pigs vaccinated intranasally using PRRS-MLV and a potent adjuvant, *M. tb* WCL substantially increased IFN-γ response associated with a greater than two-fold increase in the population of CD4 CD8 double positive T cells (T-helper/memory) in virus-challenged pigs [16]. In pigs vaccinated with an inactivated PRRSV delivered through a biodegradable nanoparticle, the frequency of IFN-γ^+^ CD4, CD8 and γδ T cell subsets were significantly enhanced and they were correlated well with the clearance of a heterologous challenged virus from the blood and lungs [33, 38]. This suggested a strong association of PRRSV clearance to IFN-γ^+^ T cell response, and therefore in this study we determined the population of various IFN-γ positive T cell subsets. In pigs, the CD4 CD8 double positive T cell was shown to possess memory and T helper phenotype, and upregulation of this activated T cell subset correlated strongly with the protective immune response against classical swine fever, Aujeszky’s disease and PRRSV [38, 45–47]. In PRRS-MLV (without adjuvant) IM received pigs, a significantly increased population of activated (IFN-γ^+^) CD4 CD8 double positive T cell was observed.

In addition, in pigs CD3ε^+^CD4α^−^CD8α^+^ bearing T cell is either a CTL or a γδ T cell, while the cell having the CD3ε^+^CD4α^−^CD8αβ^+^ phenotype is exclusively the CTL [48]. In PRRS-MLV IM (no adjuvant) vaccinated pigs, enhanced population of a T cell bearing CD3ε^+^CD4α^−^CD8α^+^ phenotypic marker was detected at 26 dpv; and activated IFN-γ^+^ CD3ε^+^CD4α^−^CD8α^+^ and γδ T cells were detected in virus challenged pigs at 14 dpc. In addition, an upregulated IFN-γ^+^ NK cell population was observed. The recall activated lymphocyte response was detected using the challenged PRRSV, but not with pooled conserved five peptides of Type 2 PRRSV of both structural and non-structural proteins [29–31], suggesting that in our study tested peptides may not be highly immunogenic in pigs. Overall, increased population of activated (IFN-γ^+^) T cell subsets in PRRS-MLV IM received pig groups compared to IN delivery was observed. However, due to low NA titers in the IM vaccinated pigs, the T cell response data alone may not suggest the heightened heterologous protective response.

Both route of vaccination and administration of immunomodulators must be considered in improving vaccine immunity [49]. Aase et al. [50] report that IM immunization with meningococcal outer membrane vesicles in humans has induced significant IgG response, opsonophagocytic activity and serum bactericidal activity compared to IN group; but IN immunization with 10× more vaccine antigen induced persistent mucosal and systemic antibody responses with antibacterial activity. In conclusion, until we identify a cost-effective potent adjuvant which has potent adjuvant effects akin to *M. tb* WCL, it is beneficial to use PRRS-MLV by IM route; which is currently practiced in most of the swine producing countries to control PRRS inspite of its ability to induce limited cross-protection.
